# Metastatic Small Cell Lung Cancer Masquerading as a Pancreatic Mass: A Case Report

**DOI:** 10.7759/cureus.64397

**Published:** 2024-07-12

**Authors:** Arjun R Gampala, Divya Minnaganti, Maddison Cooper, Jessi O'Neill-Smith, Jennifer McBride, Vinay Jahagirdar, Soheila Hamidpour

**Affiliations:** 1 Internal Medicine, University of Missouri Kansas City School of Medicine, Kansas City, USA; 2 Pathology and Laboratory Medicine, University Health Truman Medical Center, Kansas City, USA

**Keywords:** thorough differential, abdominal pathology, metastasis, pancreatic mass, small-cell lung carcinoma

## Abstract

Small cell lung cancer (SCLC) is notorious for its aggressive behavior and propensity for metastasis. Although metastasis to the pancreas from SCLC is relatively rare, it warrants attention due to its overlapping symptomatology with primary pancreatic malignancies and other abdominal pathologies (such as those involving the liver or gallbladder). Despite recent advances, the mechanisms driving SCLC metastasis to the pancreas remain elusive, providing challenges in diagnosis and treatment.

This case report details the presentation of a 59-year-old woman with SCLC metastasis to the pancreas, initially masquerading as primary pancreatic carcinoma, as highlighted by her presenting symptoms of jaundice, weight loss, and abdominal pain. Diagnostic workup, including imaging studies and tissue sampling, confirmed the unexpected presence of metastatic SCLC in the pancreas. The patient was ultimately transferred to a tertiary care facility for further workup.

This case serves as a reminder to maintain a broad differential diagnosis, particularly in the face of such an unusual presentation. It also highlights the need for further research to elucidate the molecular and cellular mechanisms driving SCLC metastasis to the pancreas, with the ultimate goal of improving diagnostic accuracy and therapeutic outcomes for patients with this aggressive disease.

## Introduction

Small cell lung cancer (SCLC) is a highly aggressive form of lung cancer characterized by rapid progression and early metastasis [1]. Despite advancements in treatment, metastatic dissemination remains a formidable challenge in managing SCLC, contributing to poor prognosis and limited treatment options [2]. The pancreas is an infrequent site of metastasis from SCLC; according to research studies, SCLC accounts for approximately 1-2% of all reported pancreatic metastases [1]. Clinically, however, the pancreas remains a significant site of metastatic consideration as it presents unique diagnostic dilemmas due to overlapping symptoms with primary pancreatic malignancies and other abdominal pathologies [1,2]. When considering differential diagnoses for pancreatic masses, it is important to recognize that while SCLC metastasizing to the pancreas is rare, other more common metastatic cancers should also be considered. These include primary tumors from the colon, stomach, and kidney, as well as melanomas and breast cancers, all of which have a higher propensity to metastasize to the pancreas. These diagnostic dilemmas lead to delays in treatment, further contributing to the low median survival of SCLC, especially in the context of metastasis.

The case under review involves a patient with SCLC metastasis to the pancreas, which manifested as an unusual presentation with jaundice, weight loss, and abdominal pain. These symptoms could easily be attributed to primary pancreatic carcinoma or other abdominal malignancies, highlighting the complexities in diagnosing metastatic SCLC to the pancreas. Proper identification and differentiation among these potential sources are crucial for accurate diagnosis and effective treatment planning.

The pancreas is considered a sanctuary site for various cancers, owing to its intricate vascular supply and lymphatic drainage patterns that facilitate both hematogenous and lymphatic dissemination of cancer cells [3]. However, the precise mechanisms driving the preferential metastasis of SCLC to the pancreas remain poorly understood [3]. Recent research has shed light on the molecular pathways implicated in SCLC metastasis, offering potential insights into therapeutic targets. Dysregulation of tumor suppressor genes such as TP53 and RB1, along with the activation of oncogenic signaling pathways like PI3K/AKT and MAPK, are thought to play critical roles in the metastatic cascade of SCLC [3,4]. Moreover, interactions within the tumor microenvironment and between cancer cells and stromal components are believed to contribute significantly to facilitating pancreatic metastasis [4].

Understanding the intricate molecular and cellular processes driving SCLC metastasis to the pancreas, in addition to recognizing the clinical presentation, is essential for improving patient outcomes in such a devastating situation.

## Case presentation

A 59-year-old woman presented to the emergency department in rural Missouri with a two-and-a-half-month history of epigastric pain. She described the pain as a dull, aching sensation with intermittent sharp episodes radiating to her back. During this period, she also developed right upper quadrant abdominal pain, jaundice, and an unintended weight loss of 30 pounds. Other noteworthy symptoms included dark urine and pale stools. The patient had assumed her dark urine was from a urinary tract infection, so she had been drinking large amounts of water daily. The patient had seen a primary care physician two weeks before presentation and, at that time, demonstrated significantly elevated liver enzymes. Her past medical history yielded no significant information pertinent to her current situation. Additionally, the patient had no significant family history other than hypertension in both parents. She had one or two alcoholic drinks on rare occasions and had smoked one pack of cigarettes per day for the last six years.

**Figure 1:**
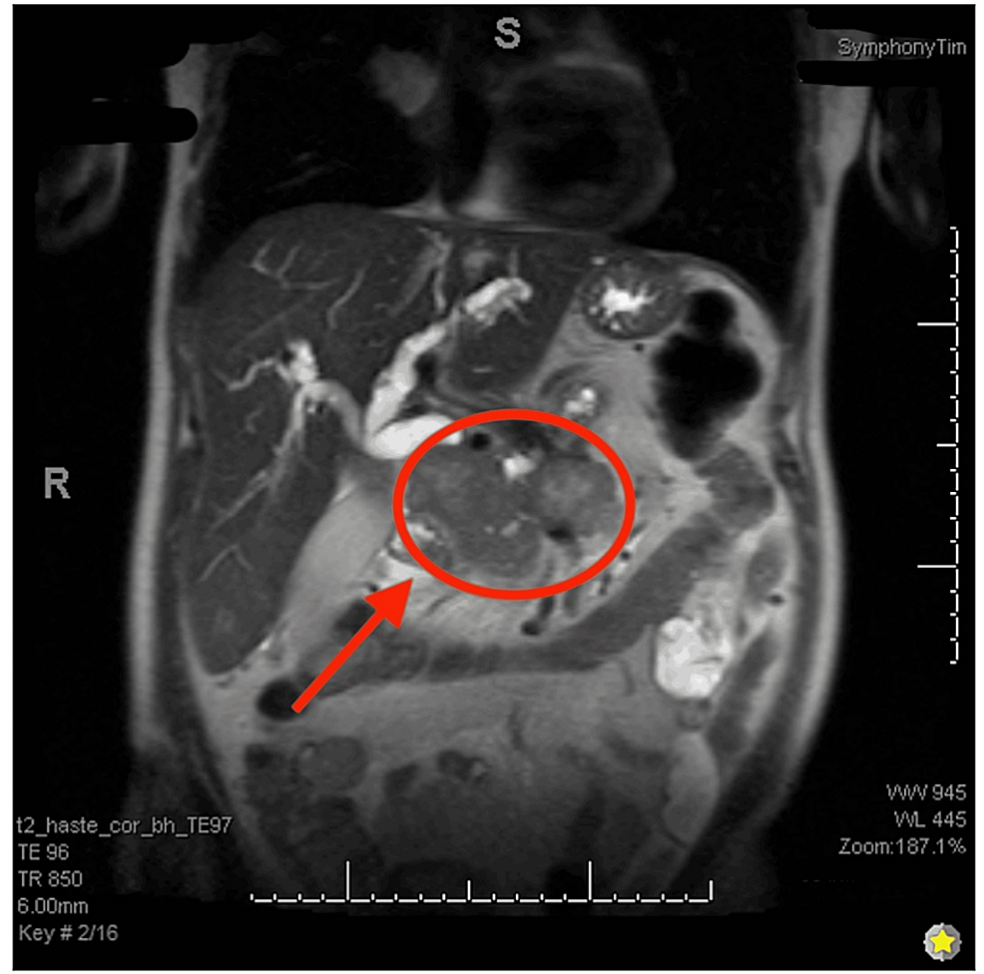
T2-weighted MRI abdomen with and without MRCP (coronal view) showing pancreatic mass post-gadolinium contrast administration. A pancreatic mass of 8.2 x 9.6 x 6.6 cm with dilation of intra and extrahepatic bile ducts. MRCP: magnetic resonance cholangiopancreatography

**Figure 2:**
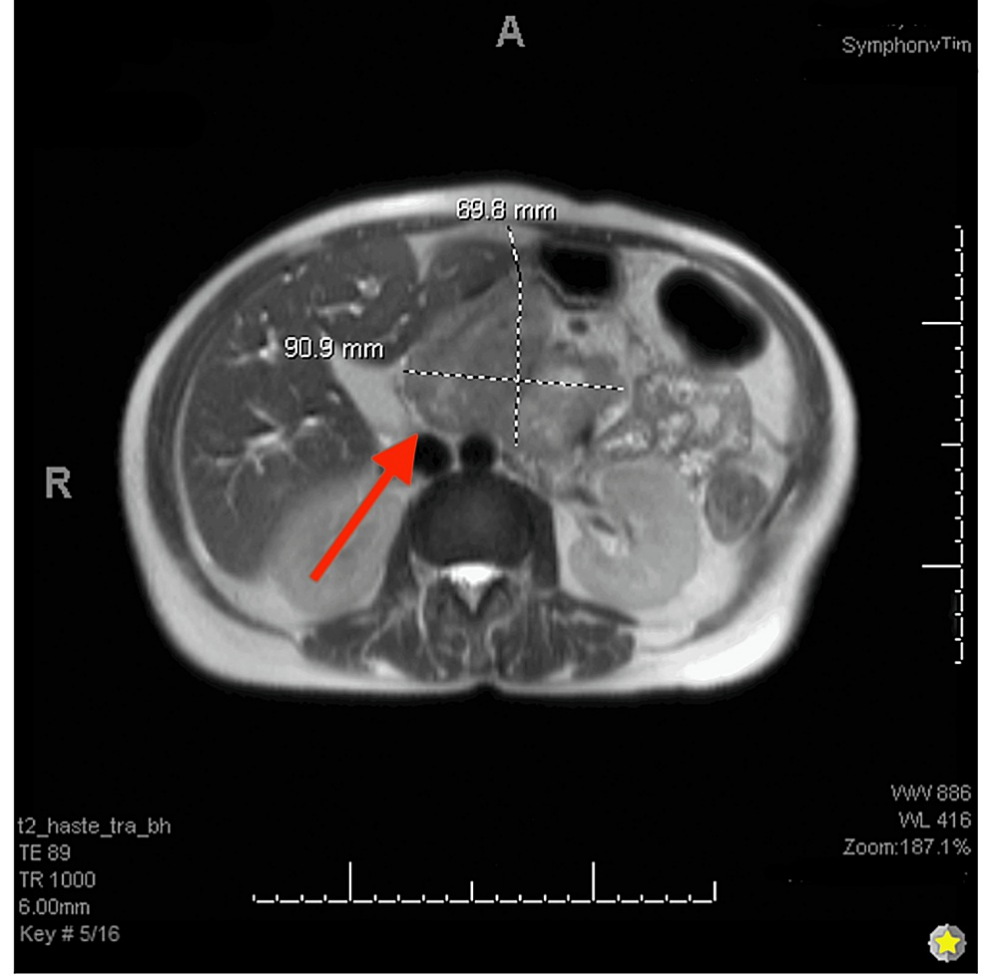
T2-weighted MRI abdomen with and without MRCP (axial view) showing pancreatic mass post-gadolinium contrast administration. MRCP: magnetic resonance cholangiopancreatography

On exam, a soft, mildly tender mass was noted in the epigastric region. The patient appeared cachectic with jaundiced skin and icteric sclera. Upon arrival, labs were significant for hypoosmolar hyponatremia, with serum osmolarity of 270 mOsm/kg (n=275 to 295 mOsm/kg) and sodium of 131 mEq/L (n=135-145 mEq/L). Other significant labs included hypokalemia of 3.0 mmol/L (n=3.6-5.2 mmol/L), total bilirubin of 20 mg/dL (n=0.1-1.2 mg/dL), direct bilirubin of 13.72 mg/dL (n=<0.3 mg/dL), lipase of 332 U/L (n=0-160 U/L), leukocytosis of 12,900/mm³ (n=4,500-11,000/mm³), hemoglobin of 10.3 g/dL (n=13.5-17.5 g/dL), aspartate aminotransferase (AST) of 172 U/L (n=0.8-48 U/L), alanine aminotransferase (ALT) of 182 U/L (n=0.7-55 U/L), and a prolonged international normalized ratio (INR) of 1.73 (n=2.0-3.0). CT abdomen/pelvis showed lobulated 7.8 cm solid enhancing mass arising in the head/uncinate process region of pancreas consistent with primary pancreatic carcinoma, associated with obstruction of the common bile duct and pancreatic duct with moderate/severe bile duct dilation. Magnetic resonance imaging (MRI) of the abdomen with and without magnetic resonance cholangiopancreatography (MRCP) was performed; these images showed a heterogeneous mass centered within the pancreatic head measuring 8.2 x 9.6 x 6.6 cm with abrupt cutoff and dilation of the intrahepatic and extrahepatic bile ducts and pancreatic duct (Figures 1-2).

**Figure 3:**
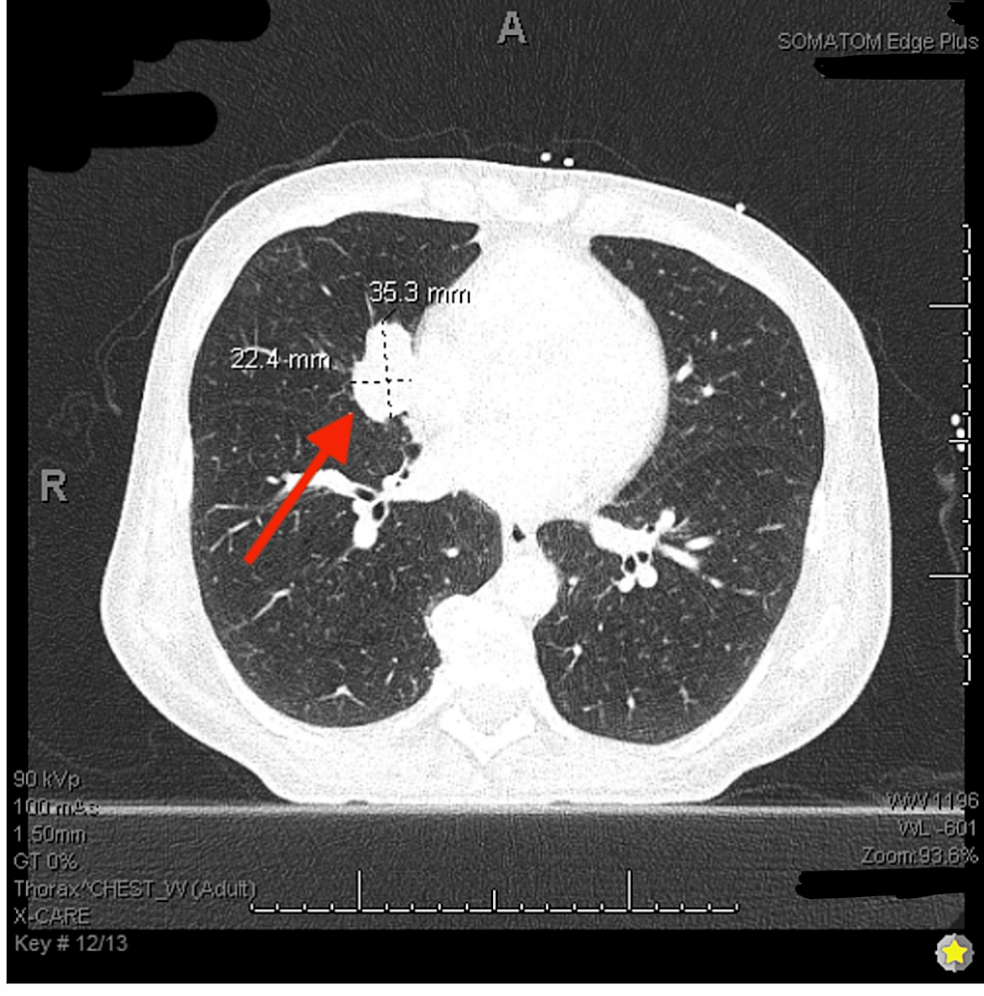
CT chest with iodine contrast showing right middle lobe lung mass of 2.2 x 3.5 cm.

**Figure 4:**
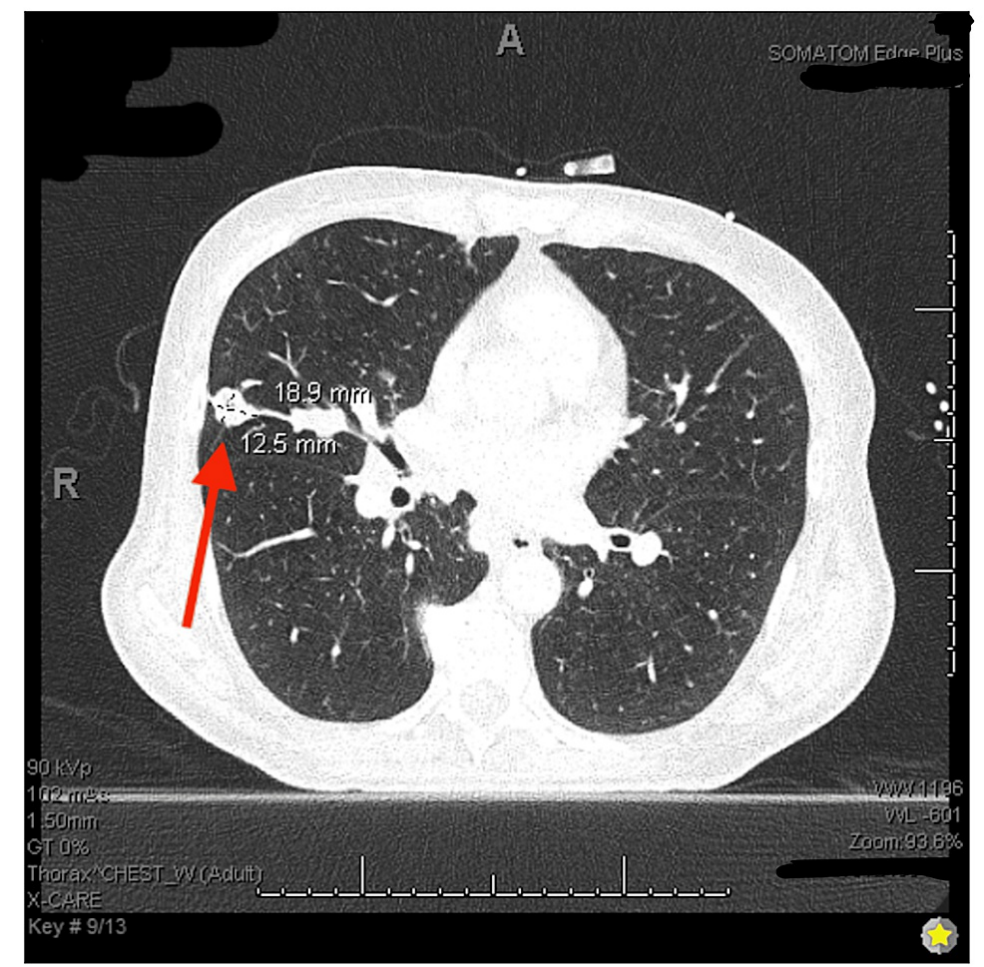
CT chest with iodine contrast showing subpleural right middle lobe lung cavitary nodule (1.3 cm x 1.9 cm lung mass).

**Figure 5:**
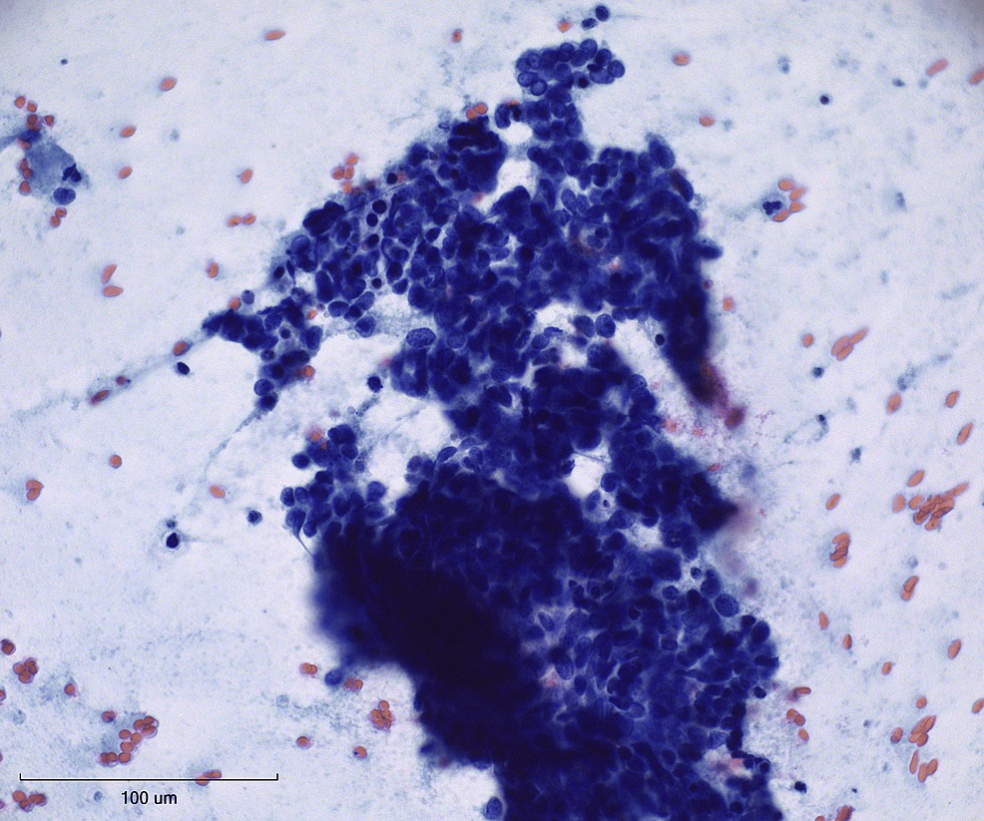
Pap stain at 40X magnification Crowded clusters of small cells exhibiting hyperchromatic nuclei and minimal cytoplasm.

**Figure 6:**
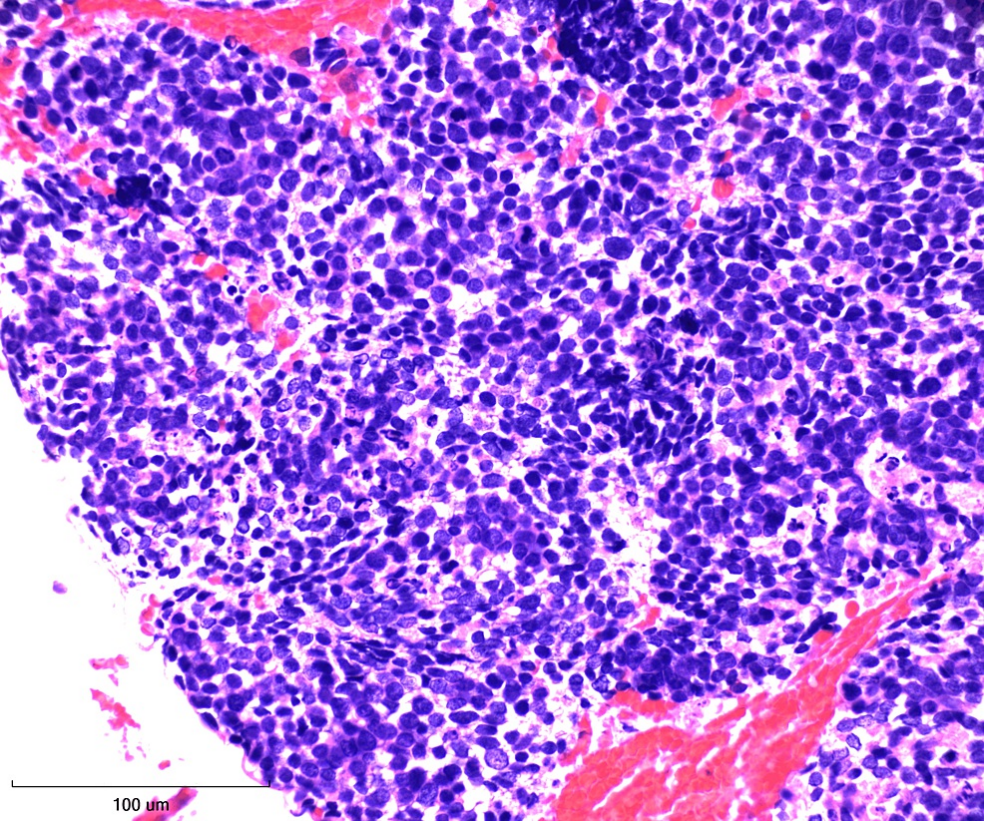
Cell block at 40X magnification with hematoxylin and eosin (H&E) stain Crowded clusters of small cells displaying fine nuclear chromatin and scant cytoplasm.

**Figure 7:**
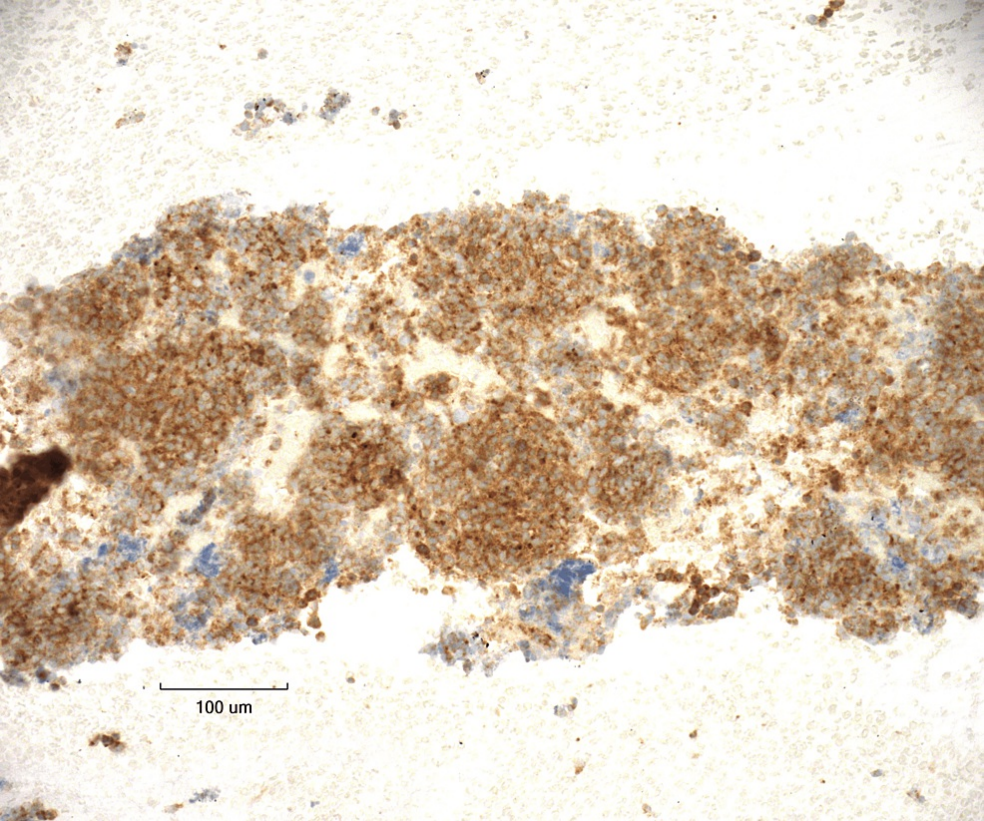
Synaptophysin stain at 20X magnification Tumor cells show cytoplasmic positivity for Synaptophysin (neuroendocrine marker).

Oncology was consulted and recommended tissue diagnosis prior to initiating treatment. During endoscopic retrograde cholangiopancreatography (ERCP) the following day, a common bile duct stricture was dilated, brushings were obtained for cytology, and stents were placed with adequate drainage, resulting in down-trending bilirubin levels. The brush cytology report was negative for malignancy. A CT chest was then obtained, showing multiple lung nodules suspicious for metastases with extensive mediastinal and hilar adenopathy (Figures 3-4). The patient underwent an endoscopic ultrasound of the upper gastrointestinal (GI) tract during hospitalization and multiple fine-needle aspirations of the pancreatic masses were obtained. Cytology smears and cell block demonstrated a hypercellular specimen with large groups of small cells with hyperchromatic nuclei with fine chromatin and scant cytoplasm (Figures 5-6). Tumor cells were positive for the immunostains pancytokeratin (epithelial marker) and synaptophysin (neuroendocrine marker) (Figure 7); and showed >90% proliferation index by Ki67 immunostain. These changes are consistent with poorly differentiated neuroendocrine tumor/small cell carcinoma. Tumor cells were also positive for TTF1 and negative for CDX2, favoring lung origin.

The patient was ultimately transferred back to a rural hospital to pursue chemotherapy and radiation treatment closer to home. She had a follow-up with gastroenterology and returned to our center two months later, where she successfully underwent the removal and exchange of a biliary stent in the lower third of the bile duct. This was the only additional medical care she received at our institution; the patient continues to attend hematology-oncology, gastroenterology, staging, and chemotherapy appointments at the rural hospital.

## Discussion

SCLC accounts for approximately 15% of lung cancers [5] and is notorious for its high mortality rate due to widespread metastasis and aggressive behavior [5,6]. Typically associated with respiratory symptoms such as dyspnea and cough, SCLC can also present with extrapulmonary manifestations including bone pain, neurological dysfunction, weight loss, and fatigue resulting from metastasis to distant sites like bone, brain, or adrenal glands [6]. Another common site of metastasis is the liver, often manifesting with jaundice due to intrahepatic cholestasis [6,7].

Pancreatic metastasis from SCLC, although relatively uncommon compared to other sites (renal, colon, stomach, kidney, breast, and melanoma), presents unique challenges in diagnosis and management due to overlapping symptoms with a spectrum of pancreatic and abdominal pathologies. However, it is crucial not to overlook the possibility of SCLC metastasis to the pancreas in patients presenting with jaundice, weight loss, and a history of heavy smoking, especially considering the poor prognosis. The five-year survival rate of SCLC is 18% for regional metastasis and 3% for distant metastasis [8]. Specifically, there is a median survival of six to eight months for pancreatic metastasis of SCLC [7]. This can be closely compared to the five-year survival rate of pancreatic cancer which is 16% for regional metastasis and 3% for distant metastasis [9]. In particular, one study found that the median survival rate for patients with metastatic pancreatic cancer was 179 days (approximately six months) [10].

Secondary pancreatic cancers are relatively rare, with pancreatic metastasis reported in 1.6-10.6% of autopsy studies [7]. SCLC is recognized as an uncommon primary source of pancreatic metastasis, with non-SCLC and renal, breast, colon, kidney, and skin cancers being more common sources of secondary pancreatic tumors [6,7].

The diagnosis of SCLC metastasis to the pancreas can be challenging, as illustrated by the unusual presentation initially suggestive of primary pancreatic carcinoma in our patient. Many patients with pancreatic metastasis are asymptomatic or have nonspecific complaints, leading to incidental findings in imaging studies like CT scans. Among symptomatic cases, jaundice or abdominal pain in the context of pancreatitis is common [7]. Histologically, SCLC in the pancreas often presents as a single localized mass in the pancreatic head [7].

In clinical practice, when evaluating a patient presenting with jaundice and potential cancer (smoking history and weight loss), primary lung cancer, primary pancreatic cancer, or other abdominal pathologies are what come to mind first. In fact, metastasis to the hepatic porta or liver parenchyma is typically prioritized before metastasis to the pancreas [7]. Nevertheless, due to overlapping symptoms and diagnostic challenges, clinicians must maintain a high index of suspicion for pancreatic metastasis of SCLC to shorten the length of presentation to diagnosis.

Several pancreatic cancer diagnosis algorithms have been proposed, and though they may differ slightly, they all aim to efficiently determine the etiology and management of pancreas masses. Specific criteria for lung metastasis evaluation are not necessarily provided on many of these algorithms, but they do include imaging and tumor marker testing to begin considering malignancy as a cause. The 2024 National Comprehensive Cancer Network (NCCN) Clinical Practice Guidelines in Oncology (NCCN Guidelines®) for pancreatic tumors recommend obtaining a CT abdomen/MRI abdomen first to determine whether or not a mass is present [11]. This can also evaluate for metastasis. Afterward, NCCN Guidelines® recommends that biopsy and tumor marking testing be performed [11]. Endoscopic ultrasound is obtained to further evaluate when no metastatic disease is present [11]. Histological staining of biopsied specimens plays a crucial role in diagnosis, as it did for the patient presented in this case. The differentiation between neuroendocrine tumors of GI tract/pancreatic origin and those of lung origin is subtle, but changes the prognosis and course of treatment. In the patient's case, the tumor's immunohistochemical profile, specifically being TTF-1 positive (associated with pulmonary tumors) and CDX2 negative (associated with GI and pancreatic tumors), strongly suggests a lung origin.

Based on the extensive workup mentioned above, further therapy is then constructed. For SCLC that has metastasized to the pancreas, a multidisciplinary approach is typically recommended. Non-pharmacological treatments include palliative care measures such as nutritional support, pain management, and psychosocial support to improve the patient's quality of life. Pharmacological treatments often involve chemotherapy, with platinum-based agents like cisplatin or carboplatin combined with etoposide being standard first-line therapies [12]. Immunotherapy, specifically immune checkpoint inhibitors such as atezolizumab or durvalumab, may also be used in combination with chemotherapy for certain patients [12]. Surgical options are generally limited due to the aggressive nature of SCLC and its propensity for early metastasis, but in rare cases where the metastatic burden is low and localized, surgical resection of pancreatic metastases might be considered [11]. These treatment modalities aim to extend survival and alleviate symptoms, though prognosis remains poor for advanced SCLC.

In summary, SCLC metastasis to the pancreas represents a clinically relevant but relatively uncommon phenomenon that necessitates careful consideration in the differential diagnosis of pancreatic masses. Continued research into the molecular mechanisms driving SCLC metastasis and the development of targeted therapeutic approaches are imperative for improving diagnostic accuracy and treatment outcomes in patients with this aggressive form of lung cancer.

## Conclusions

We have presented a case of SCLC metastasis to the pancreas in a patient presenting with epigastric pain, jaundice, and smoking history. Upon further evaluation, lung imaging showed multiple pulmonary nodules, and pathology of the discovered pancreatic mass showed tissue markers favoring a lung origin (TTF1 positive, CDX2 negative). Based on the clinical exam, the patient had a classic presentation of pancreatic adenocarcinoma, but it is important to consider other etiologies of pancreatic masses, as described in this case. Pancreatic cancers secondary to malignancy have been shown in several autopsy reports, with a few case studies showing SCLC metastasis to the pancreas. Molecular mechanisms involved in SCLC are not well understood, but dysregulation of tumor suppressor genes (e.g., TP53, RB1) and activation of oncogenic signaling pathways (e.g., PI3K/AKT, MAPK) have been shown to be involved. This case is atypical of other similar patient presentations and expands upon the potential for further research in identifying pathways and treatment modalities in SCLC metastasis to the pancreas.
